# The potential risk of spinal cord injury from pedicle screw at the apex of adolescent idiopathic thoracic scoliosis: magnetic resonance imaging evaluation

**DOI:** 10.1186/s12891-015-0766-0

**Published:** 2015-10-20

**Authors:** Shoufeng Wang, Yong Qiu, Wenjun Liu, Benlong Shi, Bin Wang, Yang Yu, Zezhang Zhu, Bangping Qian, Feng Zhu, Xu Sun

**Affiliations:** Spine Surgery, Drum Tower Hospital, Nanjing University Medical School, 321, Zhongshan Road, Nanjing, China 210008

**Keywords:** Risk, Spinal cord injury, Pedicle screw, Apex, Scoliosis correction

## Abstract

**Background:**

The pedicle screw placement in scoliosis correction at the apex underlies potential risk for neurological injury. This research is to investigate the relative position of spinal cord at the apex in idiopathic thoracic scoliosis and to explore the risk of spinal cord injury from pedicle screw at the apex.

**Methods:**

Thirty-three adolescents with thoracic scoliosis were recruited in this study. The relative position of the spinal cord in the spinal canal was calculated by measuring the distance between the spinal cord and the medial wall of the pedicle on the convex and concave side through the axial plane of the apex in T2 weighted MR image. The distance from the spinal cord to the medial wall of pedicle between concave and convex side was compared respectively. The percentage of patients was calculated according to hypothesized different space (0 mm, less than 1 mm and less than 2 mm) between medial wall of pedicle and spinal cord at the apex.

**Results:**

The average distance from the spinal cord to the medial wall of pedicle at the concave side was significantly less than that at the convex side (*p =* 0.000) of the apex in the major thoracic curves before operation. In the concave side of the apex, the percentage of patients was 39.4, 66.7, 84. 5 % in hypothesized space (0 mm, less than 1 mm and less than 2 mm) between medial wall of pedicle and spinal cord. However, in the convex side of apex, the percentage of cases was 0, 0, 3.0 % in the same hypothesized space respectively.

**Conclusions:**

The screw placement is at a higher risk of spinal cord injury on the concave side than that on the convex side of apex in thoracic curve in MRI images. The screw placement in the concave side of apex should be evaluated carefully with MRI before operation.

## Background

Neurological injury is one of the most severe complications of scoliosis correction [1–7], though thoracic pedicle screw based instrumentation is the most used technique in the treatment of scoliosis due to the consistently excellent results achieved in fixation and deformity correction [8, 9]. Safe and reproducible placement of thoracic pedicle screws was commonly dependent on a better understanding of abnormal anatomy in scoliosis. However, the incidence of screw misplacement increased up to 43 % [10, 11] when all the screws were evaluated by computed tomography (CT) postoperatively. The difficulty of thoracic pedicle screw placement in scoliosis correction underlies the potential risk for spinal cord and other important structures injury [12, 13]. Dinesh et al. [14] evaluated 261 thoracic pedicle screws (T1-T12) using intraoperative CT scan. Of which, four screws (1.5 %) breached the pedicle wall by more than 2 mm and were required immediate revision. Mac-Thiong et al. [12] reported 9 cases with pedicle screws misplaced totally with the spinal canal during posterior surgery for AIS and spinal canal intrusion varied from 21–61 % in their study. They suggested that any pedicle screw misplaced within the spinal canal should be removed because of possible early or late neurological complications. Although some new techniques, such as 3D-based navigation [15, 16] and Ball tip technique [17], had improved the accuracy of pedicle screw placement in thoracic spine, there was also a percentage of pedicle breach (< or = 2 mm were 9 %, > 2 mm were 3 %). The risk of spinal cord injury is still lying in the thoracic deformity correction with pedicle screw placement. Although no neurovascular complications were encountered, Abul-Kasim et al. [18] reported that the overall rate of screw misplacement was 17 % (*n* = 149) and 6.1 % were medially placed. However, their grading system was based on whether the cortical violation is partial or total rather than on mm-basis. Modi et al. [19] reported that the accuracy rate of thoracic pedicle screw was 89.9 % with regard to the hypothesized safe zone definition in thoracic scoliosis [8], but there was a 10.3 % medial misdisplaced rate totally. Thoracic pedicle screw fixation is potentially risky because of little space between the spinal cord and medial wall of pedicle in the concave side of apex. The incidence of screw-related neurologic complications ranges from 0 to 0.9 % [10, 11] during the treatment of spinal deformities with thoracic pedicle screws. Sarlak et al. [20] reported that the rate of medial misplaced pedicle screw was 10.8 % from a study of 1797 screws in 148 scoliosis patients. The definition of unacceptalbe screw was medial violation of greater than 2 mm. They suggested that the acceptability of medial pedicle breach may change in each level with different canal width and a different amount of cord shift.

However, to our knowledge, the studies that investigated the distance between the spinal cord and medial wall of pedicle on the convex and concave side of the apex before operation were not currently found. The purpose of this study is to investigate the relative position of spinal cord at the apex in adolescent patients with idiopathic thoracic scoliosis in supine position before operation and to explore the potential risk of spinal cord injury from placement of pedicle screws at the apex.

## Methods

Thirty-three patients with idiopathic thoracic scoliosis were included in this study. This research was approved by Ethic Committee of Nanjing University. There were 3 males and 30 females. The average age is 14.8 years old (12–19 yrs). All the patients were classified as Lenke type 1 scoliosis according to the Lenke’s classification. There were 16 patients with type 1A, three with type 1A, five with type 1B, five with type 1B, three with 1C and one with 1C. The number of apex in T8, T9, T10 and T11 was 16, 11, 4 and 2 respectively. The average Cobb’s angle of thoracic curve before operation was 49.5° (45°–71°). The classification, Cobb’s angle, distance between spinal cord and medial wall of thoracic pedicles on the convex and concave side of the apex in thoracic scoliosis before operation were summarized in detail in the Table 1.

**Table 1:** Data of spinal cord shift in adolescents with idiopathic thoracic scoliosis before and after operation

NO.	LSC	Apex	Cobb’s angle pre-op	D1 (mm)	D2 (mm)
1	lenke1B	T9	50	5.27	2.2
2	lenke1C	T9	46	7.03	0
3	lenke1A	T9	45	4.39	0.44
4	lenke1B	T9	70	6.59	0
5	lenke1B	T8	50	4.83	0
6	lenke1B	T8	45	4.39	0
7	lenke1B	T8	45	1.32	0
8	lenke1A	T10	45	8.35	3.08
9	lenke1A	T10	45	6.59	1.32
10	lenke1B	T9	46	5.71	0.88
11	lenke1A	T11	48	2.64	0.44
12	lenke1A	T8	50	7.47	2.64
13	lenke1B	T8	45	9.52	0.76
14	lenke1A	T8	45	6.15	0
15	lenke1B	T8	62	4.39	0
16	lenke1A	T8	55	3.52	0
17	lenke1A	T9	65	3.52	0
18	lenke1C	T8	45	5.27	0.44
19	lenke1A	T9	45	5.71	0.44
20	lenke1C	T8	46	4.83	0.88
21	lenke1B	T9	71	6.86	0
22	lenke1A	T10	46	4.39	2.2
23	lenke1A	T8	45	6.59	0.44
24	lenke1B	T9	45	3.43	0
25	lenke1A	T11	45	5.27	0.88
26	lenke1A	T8	45	4.39	1.76
27	lenke1A	T10	46	5.5	0
28	lenke1C	T9	58	4.12	0
29	lenke1A	T8	58	3.52	1.76
30	lenke1A	T8	45	6.47	1.9
31	lenke1A	T8	45	6.15	1.32
32	lenke1A	T9	45	6.15	1.32
33	lenke1A	T8	45	4.95	2.67

Lenke’s classification, LSC; D1: Spinal cord to medial wall of pedicle in the convex side; D2: Spinal cord to medial wall of pedicle in the concave side

**Fig. 1:**
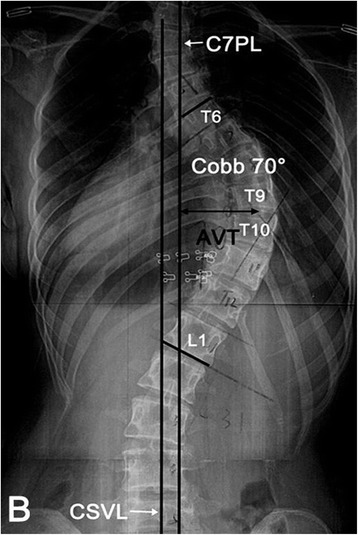
The full frontal standing radiograph of the spine in a AIS patient (right thoracic scoliosis, a Cobb’s angle 70°, Lenke 1B). The measurements include Cobb’s angle, apex and apical vertebrae translation. AVT represents the distance between apex or apical disc and line C7PL

**Fig. 2:**
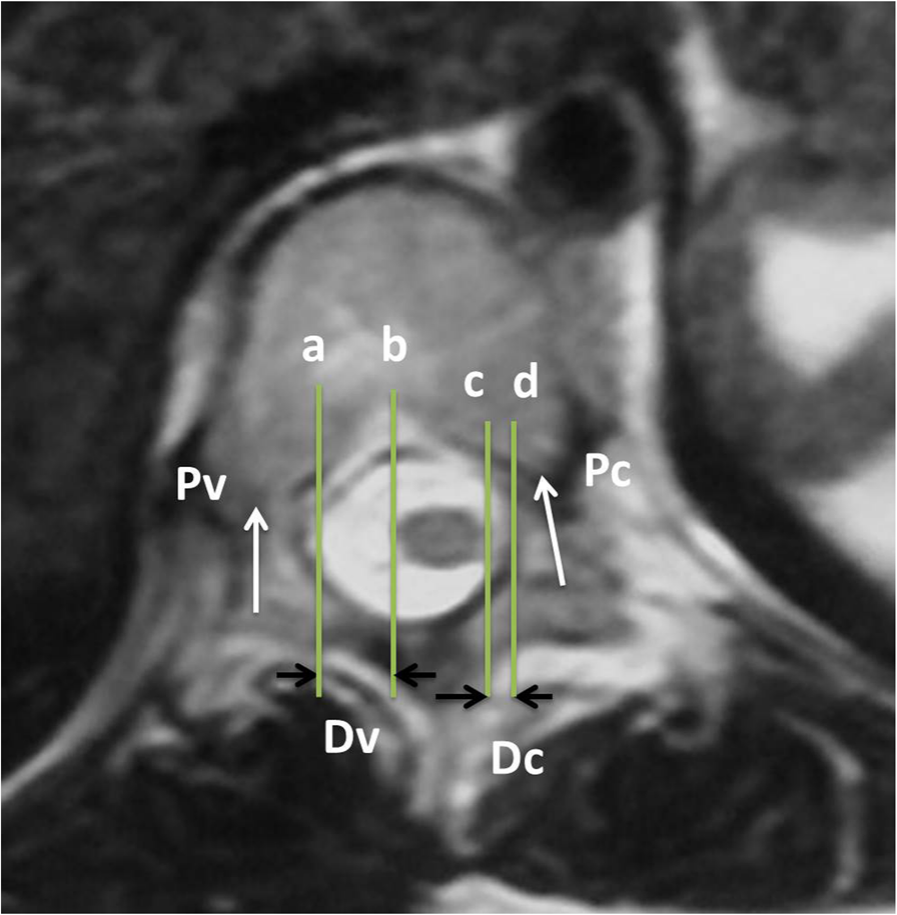
The measurement of the shift of spinal cord. The transverse plane of apex (T8) in MRI with T2 weighted imaging for a female with idiopathic scoliosis (right thoracic scoliosis, a Cobb’s angle 46°, Lenke 1C). Pv and Pc represent direction of pedicle on the convex and concave side of apex. Line a and b, line c and d were level with Pv and Pc respectively. Line a and b were tangents of medial wall of pedicle and spinal cord on the convex side of the apex. Line c and d were tangents of medial wall of pedicle and spinal cord on the concave side of the apex. The vertical distance between line a and b (Dv) and line c and d (Dc) represented distance between spinal cord and medial wall of pedicle on the convex and concave side respectively

All patients and parents of the patients under 16 years old signed the informed consent form. They all had frontal and lateral standing radiographs of the total spine (Fig. 1). Axial T2-weighted images were obtained using a 1.5-T MRI system (Philips Gyroscan Intera Master, Eindhoven, Netherlands). The measurements were performed by PACS system (First Tech Company, USA). The distance was measured on the PACS software with a linear distance tool. The image of section which was perpendicular to the longitudinal axis of the apex was selected for the measurement. The mid-axial sections were chosen. The distance between the spinal cord and concave and convex pedicles was measured in the same slice for the same level. The measurement was done consistently with a standard measuring scale provide by the PACS system software. As shown in Fig. 2, Pv (convex pedicle, Pv) and Pc (concave pedicle, Pc) represented the axial direction of pedicle on the convex and concave side of the apex respectively. Line A and Line D were tangent to the medial walls of the pedicle. Line B and C were tangent to the outer edge of the spinal cord. All lines were parallel to the pedicle direction. The vertical distance between Line A and Line B (Dv) and Line C and Line D (Dc) represented distance between the spinal cord and the medial wall of pedicle on the convex and concave side respectively. Two spine fellows measured all distances independently and means of distances between the spinal cord and medial wall of concave and convex pedicle at the apex were calculated to reduce interobserver errors (R = 0.91 for convex side, R = 0.93 for concave side; Pearson correlation coefficient) and intraobserver errors (R = 0.925 to 0.947; Pearson correlation coefficient).

**Fig. 3:**
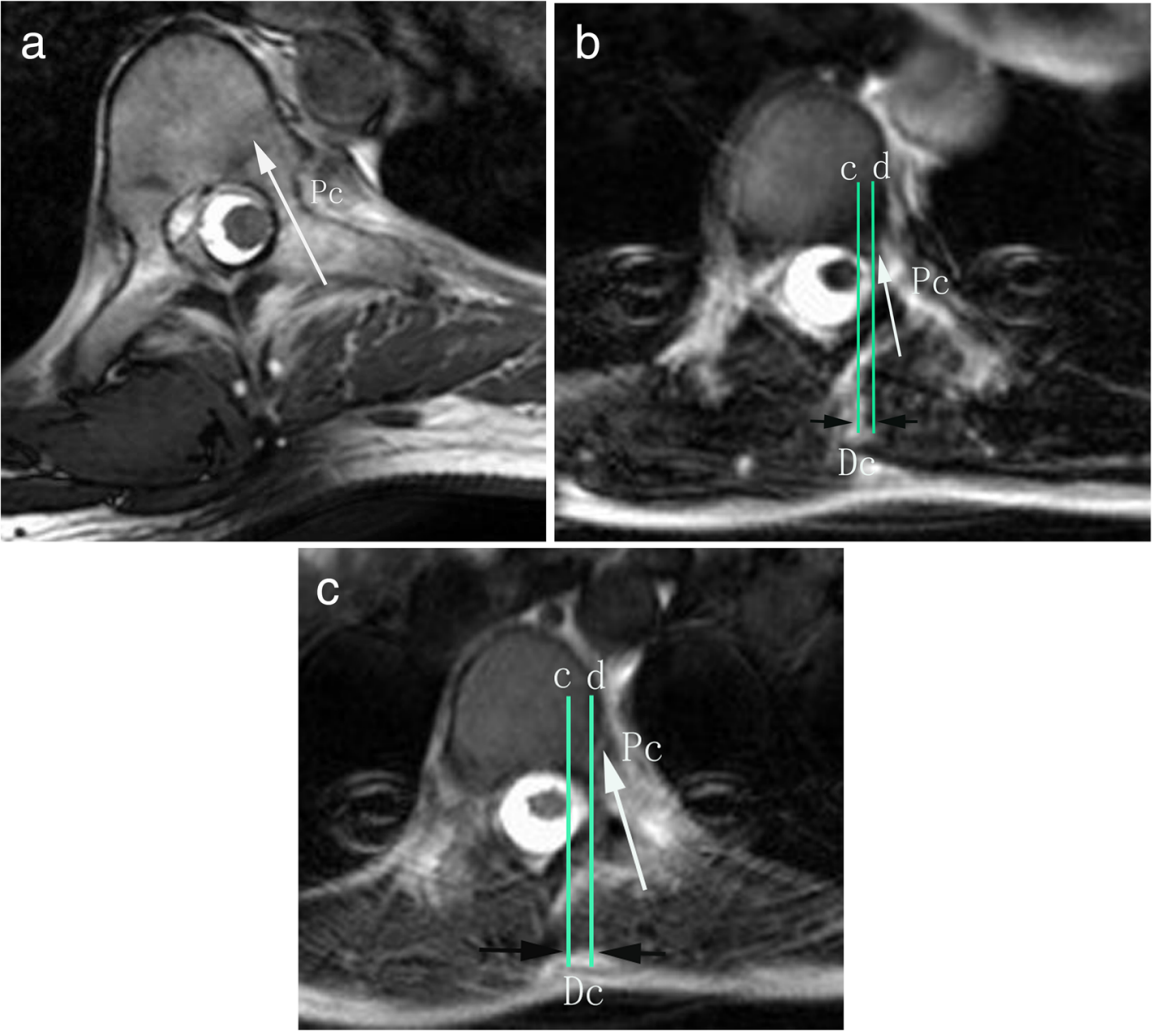
The measurement of the shift of spinal cord. The vertical distance between line c and d (Dc) represented distance between spinal cord and medial wall of pedicle on the concave side with 0 mm (**a**), 0.88 mm (**b**) and 1.9 mm (**c**)

According to different distance (0 mm, less than 1 mm and less than 2 mm) (Fig. 3a, b and c) between medial wall of pedicle and spinal cord in the apex before operation, the percentage of patients was calculated.

All the data were collected in a computerized database. The distance from the spinal cord to the medial wall of pedicle was recorded and was compared between the convex side and concave side. All comparisons were performed by the student *T* test. The data was analyzed by the SPSS software 13.0 (SPSS Inc. Chicago, IL, USA). Statistical significance was *P* < 0.05.

## Results

In the supine position, the distance from the spinal cord to medial wall of the pedicle was 0.84 ± 0.95 mm and 5.31 ± 1.66 mm on the concave and convex side respectively. The distance on the concave side was significantly less than that on the convex side (*p =* 0.000) of the apex in the thoracic curves before operation (Table 2).

**Table 2:** Comparison of distance from spinal cord to the medial all of the pedicle between convex side and concave side before operation

Distance from spinal cord to the medial wall of pedicle	Convex side (Mean ± SD)	Concave side (Mean ± SD)	*P*
	5.31 ± 1.66 mm	0.84 ± 0.95 mm	0.000*

*Statistically significant

On the concave side of apex, the percentage of patients was 39.4, 66.7 and 84.5 % in hypothesized space (0 mm, less than 1 mm and less than 2 mm) between medial wall of pedicle and spinal cord before operation respectively. On the convex side of apex, the percentage of cases was 0, 0 and 3.0 % in the same hypothesized space before operation respectively (Table 3).

**Table 3:** The percentage of patients according to space between pedicle medial wall and spinal cord

Space (mm)	D1(33)	D2(33)
0 mm	0 %	39.4 %
<1 mm	0 %	66.7 %
<2 mm	3.0 %	84.5 %

D1: Spinal cord to medial wall of pedicle in the convex side before operation; D2: Spinal cord to medial wall of pedicle in the concave side before operation

## Disscussion

Iatrogenic spinal cord injury was one of the most severe complications of scoliosis correction. The incidence of neurological complications for spinal deformity surgery had been reported by the Scoliosis Research Society as less than 1 %. The placement of pedicle screws in the thoracic spine for treatment of pediatric deformity had been reported to be safe despite the high rate of malpositioned screws. But, neurologic complication was encountered and reported. Hicks et al. [21] reported 7 pedicle screw complications over a 17 years experience of treating adolescent idiopathic scoliosis including thoracic pain with radiculopathy, somatosensory-evoked potentials disappearance after screw insertion, Brown Sequard syndrome, paraplegia and catastrophic neurologic events. There were other 3 studies which reported dural leaks during screws placement [11, 22, 23]. The pedicle screws are often applied at the apex because of the better correction strength. However, there were few investigations which reported how safe it was when the pedicle screw implanted in the apex because of the small space between medial wall of pedicle and spinal cord on concave side of apex in MRI images before scoliosis correction.

In the present study, the distance from spinal cord to medial wall of pedicle on the concave side was significantly less than that on the convex side (*p =* 0.000) of the apex in thoracic scoliosis before operation. The percentage of patients was 39.4, 66.7 and 84.5 % in hypothesized space (0 mm, less than 1 mm and less than 2 mm) between medial wall of pedicle and spinal cord on the concave side of apex respectively. However, in the convex side of apex, the percentage of cases was 0, 0 and 3.0 % in the same hypothesized space before operation respectively. This suggested that there was a smaller space between medial wall of pedicle and spinal cord in the concave side of the apex in MRI images. The misplaced pedicle screw on the concave side was more dangerous to spinal cord than that on the convex side.

Expect for the small spaces between spinal cord and the medial wall of the pedicle on the concave side, the pedicles of the thoracic vertebrae were smaller than those in the lumbar spine and there was a relative increase of injury to neurologic structures theoretically. Vishal et al. [24] investigated the abnormal pedicles in spines with AIS and normal cases. They found that a significantly higher prevalence of abnormal pedicles in the patients with AIS and most of the abnormal pedicles were in thoracic spine, on the concave side, and in the periapical and apical regions. This risk was further increased because of slimmer, more distorted, more sclerotic and shorter pedicles in thoracic scoliosis [25]. Three hypothesized different zones on the medial encroachment with CT scanning proposed by Kim et al. [8] were as follows, a “definite safe zone” (<2 mm),“probable safe zone” (2–4 mm), and “questionable safe zone” (4–8 mm). In their study, 10 screws were with medial cortical wall violation (between 2.5 and 5.0 mm). No perioperative complications and postoperative neurologic complications were encountered. The authors also assumed that this encroachment was not occurring at the concave apex of a scoliosis deformity where these tolerances might not be acceptable, which was in agreement with the result of this study.

However, Polly et al. [26] performed a volumetric analysis of canal intrusion of pedicle screws and hook. They concluded that accounting for the “screw shadow,” a thoracic pedicle screw must have a medial perforation of > 2 mm to approach the same intrusion volume. Without direct neural injury, this provided the evidence of the clinical finding of medial perforation of up to 4 mm without neurologic compromise which was larger than what we found by the MRI in this study. This suggested that the space between the spinal cord and medial wall of concave pedicle at the apex should be evaluated carefully not only by MRI but also by CT and the other approaches before the operation. On the other hand, the cases with neurological deficit do not always have misplacements in the periapical region. Therefore, even the small spaces between spinal cord and medial wall of concave pedicle at the apex exists, there is some possibility to place the pedicle screw in the concave pedicle correctly without any complications. Currently, Vallespir et al. [27] invented the technique of vertebral coplanar alignment (VCA) in which the thoracic scoliosis correction began in the convex side with pedicle screws placement. As it had been mentioned before [28], pedicles on the convex side presents some advantages when compared with those on the concave side. One of the advantages was that the relatively increased “safe zone” for pedicle screw insertion on the convex side which implies that a medial pedicle screw penetration on the convex side might be tolerated better without injury to the spinal cord than a penetration on the concave side of a scoliotic curve, especially at the apex of the curve [29]. This was in agreement with our study that the distance from spinal cord to medial wall of pedicle on the convex side was significantly more than that on the concave side of the apex in thoracic scoliosis.

However, in this study, we just investigated the axial plane between concave side and convex side without considering the ventral and dorsal shifting and rotation of spinal cord before operation. We did not investigate the distance between the spinal cord and medial wall of concave pedicle of each vertebra within the whole Cobb’s angle. The other limitation is that we did not investigate the confined anatomical dimensions of the concave pedicles such as lesser diameters and more obliquely oriented due to vertebral rotation. As we all know, the risk of neurological injury with thoracic pedicle screw insertion on the concave side is also dependent on the anatomy of these pedicles. In order to improve the accuracy of pedicle screw placement and decrease the risk of neurological injury at the apex, surgeons should find a better way what they like to use during the operation such as the intraoperative CT, radiofluroscopy, or making intraoperative laminotomies with direct medial wall palpation of the pedicle to assure that the screws are not penetrating medially into the spinal canal.

## Conclusions

The screw placement is at a higher risk of spinal cord injury on the concave side than that on the convex side of apex in thoracic curve in MRI images. The screw placement in the concave side of apex should be evaluated carefully with MRI before operation.

## Abbreviations

Pv: Convex pedicle
Pc: Concave pedicle
VCA: Vertebral coplanar alignment
AIS: Adolescent idiopathic scoliosis
LSC: Lenke’s classification

## References

[1] Been HD, Kalkman CJ, Traast HS, de Ongerboer Visser BW (1994). Neurologic injury after insertion of laminar hooks during Cotrel-Dubousset instrumentation. Spine.

[2] Grossfeld S, Winter RB, Lonstein JE, Denis F, Leonard A, Johnson L (1997). Complications of anterior spinal surgery in children. J Pediatr Orthop.

[3] Mooney JF, Bernstein R, Hennrikus WL, MacEwen GD (2002). Neurologic risk and management in scoliosis surgery. J Pediatr Orthop.

[4] Orchowski J, Bridwell KH, Lenke LG (2005). Neurological deficit from a purely vascular etiology after unilateral vessel ligation during anterior thoracolumbar fusion of the spine. Spine.

[5] Schulte TL, Lerner T, Berendes E, Bürkle H, Kiefer R, Hackenberg L (2004). Transient hemiplegia in posterior instrumentation of scoliosis. Spine.

[6] Shufflebarger HL, Grimm JO, Bui V, Thomson JD (1991). Anterior and posterior spinal fusion. Staged versus same-day surgery. Spine.

[7] Winter RB (1997). Neurologic safety in spinal deformity surgery. Spine.

[8] Samdani AF, Ranade A, Saldanha V, Yondorf MZ (2004). Free hand pedicle screw placement in the thoracic spine: is it safe?. Spine.

[9] Liljenqvist UR, Halm HF, Link TM (1997). Pedicle screw instrumentation of the thoracic spine in idiopathic scoliosis. Spine.

[10] Belmont PJ, Klemme WR, Dhawan A, Polly DW (2001). In vivo accuracy of thoracic pedicle screws. Spine.

[11] Suk SI, Kim WJ, Lee SM, Kim JH, Chung ER (2001). Thoracic pedicle screw fixation in spinal deformities: are they really safe?. Spine.

[12] Mac-Thiong JM, Parent S, Poitras B, Joncas J, Hubert L (2013). Neurological outcome and management of pedicle screws misplaced totally within the spinal canal. Spine.

[13] Ogura Y, Watanabe K, Hosogane N, Toyama Y, Matsumoto M (2013). Acute respiratory failure due to hemothorax after posterior correction surgery for adolescent idiopathic scoliosis: a case report. BMC Musculoskelet Disord.

[14] Dinesh SK, Tiruchelvarayan R, Ng I (2012). A prospective study on the use of intraoperative computed tomography (iCT) for image-guided placement of thoracic pedicle screws. Br J Neurosurg.

[15] Allam Y, Silbermann J, Riese F, Greiner-Perth R (2013). Computer tomography assessment of pedicle screw placement in thoracic spine: comparison between free hand and a generic 3D-based navigation techniques. Eur Spine J.

[16] Ughwanogho E, Patel NM, Baldwin KD, Sampson NR, Flynn JM (2012). Computed tomography-guided navigation of thoracic pedicle screws for adolescent idiopathic scoliosis results in more accurate placement and less screw removal. Spine.

[17] Watanabe K, Matsumoto M, Tsuji T, Ishii K, Takaishi H, Nakamura M (2010). Ball tip technique for thoracic pedicle screw placement in patients with adolescent idiopathic scoliosis. J Neurosurg Spine.

[18] Abul-Kasim K, Ohlin A, Strömbeck A, Maly P, Sundgren PC (2010). Radiological and clinical outcome of screw placement in adolescent idiopathic scoliosis: evaluation with low-dose computed tomography. Eur Spine J.

[19] Modi H, Suh SW, Song HR, Yang JH (2009). Accuracy of thoracic pedicle screw placement in scoliosis using the ideal pedicle entry point during the freehand technique. Int Orthop.

[20] Sarlak AY, Tosun B, Atmaca H, Sarisoy HT, Buluç L (2009). Evaluation of thoracic pedicle screw placement in adolescent idiopathic scoliosis. Eur Spine J.

[21] Hicks JM, Singla A, Shen FH, Arlet V (2011). Complications of Pedicle Screw Fixation in Scoliosis Surgery A Systematic Review. Spine.

[22] Di Silvestre M, Bakaloudis G, Lolli F, Vommaro F, Martikos K, Parisini P (2008). Posterior fusion only for thoracic adolescent idiopathic scoliosis of more than 80 degrees: pedicle screws versus hybrid instrumentation. Eur Spine J.

[23] Di Silvestre M, Parisini P, Lolli F, Bakaloudis G (2007). Complications of thoracic pedicle screws in scoliosis treatment. Spine.

[24] Sarwahi V, Sugarman EP, Wollowick AL, Amaral TD, Lo Y, Thornhill B (2014). Prevalence, distribution, and surgical relevance of abnormal pedicles in spines with adolescent idiopathic scoliosis vs no deformity: a CT-based study. J Bone Joint Surg Am.

[25] Cui G, Watanabe K, Hosogane N, Tsuji T, Ishii K, Nakamura M (2012). Morphologic evaluation of the thoracic vertebrae for safe free-hand pedicle screw placement in adolescent idiopathic scoliosis: a CT-based anatomical study. Surg Radiol Anat.

[26] Polly DW, Potter BK, Kuklo T, Young S, Johnson C, Klemme WR (2004). Volumetric spinal canal intrusion: a comparison between thoracic pedicle screws and thoracic hooks. Spine.

[27] Vallespir GP, Flores JB, Trigueros IS, Sierra EH, Fernández PD, Olaverri JC (2008). Vertebral coplanar alignment: a standardized technique for three dimensional correction in scoliosis surgery: technical description and preliminary results in Lenke type 1 curves. Spine.

[28] Qiu Y, Zhu F, Wang B, Yu Y, Zhu Z, Qian B (2011). Comparison of surgical outcomes of LenkeType 1 idiopathic scoliosis vertebral coplanar alignment versus derotation technique. J Spinal Disord Tech.

[29] Porter RW (2001). Can a short spinal cord produce scoliosis?. Eur Spine J.

